# Early-life house dust mite aeroallergen exposure augments cigarette smoke-induced myeloid inflammation and emphysema in mice

**DOI:** 10.1186/s12931-024-02774-6

**Published:** 2024-04-13

**Authors:** Nok Him Fung, Quynh Anh Nguyen, Catherine Owczarek, Nick Wilson, Nadeem Elahee Doomun, David De Souza, Kylie Quinn, Stavros Selemidis, Jonathan McQualter, Ross Vlahos, Hao Wang, Steven Bozinovski

**Affiliations:** 1 Centre for Respiratory Science & Health, School of Health & Biomedical Sciences, RMIT University, Melbourne, Australia; 2 Research and Development, CSL Limited, Bio21 Institute, Melbourne, Australia; 3 Metabolomics Australia, Bio21 Institute, University of Melbourne, Melbourne, Australia

**Keywords:** Asthma, COPD, ACO, MMP-12, Macrophages, Emphysema, T2 inflammation, IL-33

## Abstract

**Background:**

Longitudinal studies have identified childhood asthma as a risk factor for obstructive pulmonary disease (COPD) and asthma-COPD overlap (ACO) where persistent airflow limitation can develop more aggressively. However, a causal link between childhood asthma and COPD/ACO remains to be established. Our study aimed to model the natural history of childhood asthma and COPD and to investigate the cellular/molecular mechanisms that drive disease progression.

**Methods:**

Allergic airways disease was established in three-week-old young C57BL/6 mice using house dust mite (HDM) extract. Mice were subsequently exposed to cigarette smoke (CS) and HDM for 8 weeks. Airspace enlargement (emphysema) was measured by the mean linear intercept method. Flow cytometry was utilised to phenotype lung immune cells. Bulk RNA-sequencing was performed on lung tissue. Volatile organic compounds (VOCs) in bronchoalveolar lavage-fluid were analysed to screen for disease-specific biomarkers.

**Results:**

Chronic CS exposure induced emphysema that was significantly augmented by HDM challenge. Increased emphysematous changes were associated with more abundant immune cell lung infiltration consisting of neutrophils, interstitial macrophages, eosinophils and lymphocytes. Transcriptomic analyses identified a gene signature where disease-specific changes induced by HDM or CS alone were conserved in the HDM-CS group, and further revealed an enrichment of *Mmp12*, *Il33* and *Il13*, and gene expression consistent with greater expansion of alternatively activated macrophages. VOC analysis also identified four compounds increased by CS exposure that were paradoxically reduced in the HDM-CS group.

**Conclusions:**

Early-life allergic airways disease worsened emphysematous lung pathology in CS-exposed mice and markedly alters the lung transcriptome.

**Supplementary Information:**

The online version contains supplementary material available at 10.1186/s12931-024-02774-6.

## Introduction

Asthma and chronic obstructive pulmonary disease (COPD) are chronic lung diseases characterised by airflow obstruction that can exhibit distinct pathological and immunological features. COPD is generally characterised by fixed airflow obstruction caused by emphysema, chronic bronchitis and/or fibrotic remodelling of the distal airways [1], whereas asthma is characterised by reversible airways hyper-reactivity that is highly responsive to inhaled corticosteroids and bronchodilators [2]. These clinical features may overlap in patients, termed as asthma-COPD overlap (ACO), which complicates diagnosis and treatment. It is now evident that certain COPD patients can present with high sputum or blood eosinophils, and COPD can develop more rapidly in patients with a history of asthma or atopy [3].The co-existence of asthma and COPD features is associated with higher rates of exacerbations and hospitalisations and worse health-related quality of life (HRQoL) compared to asthma or COPD alone [4–6].

It is unclear how ACO originates, but current research is supportive of the widely recognized ‘Dutch hypothesis’ that asthma and COPD may share a common origin. Longitudinal studies have identified childhood asthma as an important risk factor for developing COPD later in life [7–9], where early-life impairment of lung function and male sex have been identified to be the most significant predictors of abnormal lung growth and lung function decline into early adulthood [9]. In the Tasmanian cohort, it was found that individuals in the lowest FEV1/FVC quartile at age 7 were much more likely to develop COPD (odds ratio = 5.76) and ACO (odds ratio = 16.3) at age 45 [7]. In the Childhood Asthma Management Program (CAMP) cohort, the age at which a heightened risk of COPD became apparent was as early as 26 [9]. Hence, adverse events such as childhood asthma or prematurity can predispose individuals to an accelerated and persistent decline in lung function into adulthood resulting in a more aggressive form of COPD [10]. Nonetheless, since the clinical development of COPD is multifactorial, a direct causal relationship is yet to be established. Several pre-clinical studies have combined established animal models of COPD, such as exposure to CS, and allergic asthma, such as ovalbumin or HDM sensitisation/challenge to model ACO, as summarized by Tu et al [11]. Yet, the consequence of chronic cigarette smoke exposure in the presence of atopic disease established during early life has not been explored. Furthermore, while these studies capture important pathophysiological aspects of both asthma and COPD, they typically focused on asthma severity and airway hyper-reactivity rather than emphysema development [11–17].

In this study, the primary aim was to investigate the impact of childhood asthma on COPD development following cigarette smoking through adolescence into adulthood and to uncover the underlying pathophysiological mechanisms via transcriptomic analysis. Metabolomic screening was further performed on BAL-fluid for biomarker identification as exhaled breath condensate (EBC) markers of chronic lung diseases are emerging [18]. Our study found that mice challenged with HDM aeroallergen early in life developed worse emphysema upon CS exposure in adulthood. RNA sequencing (RNA-Seq) analysis further revealed a gene signature consistent with a pathogenic MMP-12 enriched macrophage population that expanded in response to the combination of increased type 2 mediators (IL-4, IL-13 and IL-33) and CS exposure.

## Materials and methods

### Animal experimentation

All animal experiments were approved at RMIT University (AEC#24454) in accordance with the National Health and Medical Research Council of Australia (NHMRC) and ARRIVE guidelines. Male mice were used in this study as longitudinal clinical studies have shown that male sex and childhood asthma were the most significant predictors of abnormal lung function decline later in life [9]. Age matched 3-week-old male C57BL/6 mice were purchased from Animal Resource Centre (Perth, Australia). Mice were first sensitised to house dust mite extract (HDM [D. Pteronyssinus], Stellergenes Greer, US; 100 µg/35 µL) or instilled with saline (SAL) intranasally, which was followed by 4 consecutive daily challenge of HDM (25 µg/35 µL). After the sensitisation period, HDM (25 µg/35µL) was administered once weekly for 8 weeks to maintain chronic allergic airway disease. During this period, mice were also exposed to the smoke of 9 cigarettes/day (CS) or room air as described previously [19].

### Tissue collection

Mice were separated into two cohorts for tissue collection (*n* = 8 per group in both cohorts) and were culled at the end of the protocol via pentobarbital overdose (i.p., 240 mg/kg). For the first cohort of mice, bronchoalveolar lavage (BAL) was performed by flushing the lungs with ice-cold PBS using a 21G canula inserted in the trachea and whole lungs were then collected by carefully removing the trachea and connective tissues. The right superior lobe was prepared for flow cytometry immediately, and the remaining lobes were snap-frozen in liquid nitrogen and subsequently stored at -80 °C. For the second cohort of mice, lungs were inflated with 10% neutral buffered formalin (NBF) at a constant hydrostatic pressure of 25 cm for a minimum of 20 mins. The inflated lungs were excised and further fixed for another 24 h by immersion in NBF with trachea tied.

### BAL differential cell count

Total viable cells collected from BAL were calculated using a haemocytometer. Cytospin was then performed, and the slides were stained using the Hemacolor® Rapid Staining Kit (Sigma-Aldrich, US) for differential cell counting [20, 21]. The remaining fluid was centrifuged, and the supernatant (cell-free BAL fluid) was collected and stored at -80 °C for volatile organic compound (VOC) analysis.

### Histological assessment of emphysema

Cross sections of the lungs were prepared and stained with haematoxylin and eosin (H&E). Mean linear intercept (L_m_) analysis was performed on H&E-stained lung sections that were imaged on an Olympus slide scanner VS120-SS (Olympus, Japan) to determine and quantify emphysema. Five randomly selected fields, at 20× magnification, in the distal regions of each lung section were analysed. One 10 × 10 square grid, with each small square measuring 100 μm × 100 μm, was created and overlaid on an area in each field that avoids the vasculature and airways. The number of alveolar walls intersecting each horizontal grid line was then counted. The L_m_ was calculated by first subtracting the distance on each horizontal line occupied by any blood vessels and airways from the total length of all horizontal grid lines, then dividing the remaining distance by the total number of alveolar surface intersections counted. The average L_m_ across all 5 grids was used as the final L_m_ of each lung sample.

### RNA extraction, cDNA conversion and RT-qPCR

Total RNA was extracted from crushed fresh frozen lung tissue using a RNeasy kit according to manufacturer’s instructions (Qiagen, Germany). RNA was then converted to cDNA using a High-Capacity RNA-to-cDNA™ kit (Life Technologies, US). Real time quantitative polymerase chain reaction was then carried out on the Quantstudio™ 7 PCR system (Life Technologies, US) on cDNA samples using the TaqMan™ Fast Advanced Master Mix (Life Technologies, US) with the appropriate primers. Genes were normalised against Gaphd via the delta-delta Ct method as described previously [22, 23].

### Flow cytometry

The right superior lung lobes were excised and digested in Liberase TM (Sigma-Aldrich, US) at 37℃ for 45 mins on a shaking incubator. Digested tissue was then passed 5 times through a 21G needle and cells were pelleted by centrifugation at 4 ℃ for 5 mins. Red blood cells were lysed by incubating samples in ACK lysis buffer for 1 min at room temperature, followed by dilution with 10 mL HBSS. Single cell suspension was then obtained by filtering the samples through a pre-wetted 70 μm cell strainer into a 50 mL tube. Spleen samples were isolated by mechanically disrupting the tissue using a syringe plunger on a 70 µM filter and washing with HBSS. Red blood cells were lysed by incubating samples in ACK lysis buffer for 1 min at room temperature, followed by dilution with 10 mL HBSS.

For the myeloid cells, single cell suspensions of lung cells were first blocked with a rat anti-mouse CD16/CD32 antibody (Life Technologies, US) to inhibit non-antigen binding of immunoglobulins to Fc receptors before stained in Fixable Viability Dye (Life Technologies, US) and specific antibodies consisting of PE/Dazzle 594 – CD11b, BV650 – CD11c, AlexaFluor700 – CD45, PE/Cy7 – CD64, AlexFluor488 – Ly6C, BV785 – Ly6G, APC – MerTk, PerCp/Cy5.5 – MHCII (BioLegend, US), PE – Siglec F and BV711 - CD49b (BD Biosciences, US) to analyse leukocyte subsets. Stained cells were fixed using an eBioscience™ IC Fixation kit (Life Technologies, US) and analysed on a BD LSRFortessa™ Flow Cytometer (BD Biosciences, US). A strict gating strategy was used to determine different immune cell populations in single viable cells.

For the lymphoid cells, single cell suspensions of lung and spleen cells were first blocked with a rat anti-mouse CD16/CD32 antibody (Life Technologies, US) before staining in Fixable Viability Dye Near InfraRed (Life Technologies, US) and specific antibodies consisting of PerCP-Cy5.5 –CD3, V450 – CD8, FITC – CD4, PE-Cy7 – CD44 and SB600 – CD62L, to analyse T cell subsets. Stained cells were analysed on a BD LSRFortessa™ Flow Cytometer (BD Biosciences, US).

### Volatile organic compound analysis

Snap frozen BALF was thawed on ice. A 450 µL aliquot was transferred into a 20 mL vial and 4 µL of acenaphthene-d10 (concentration 2 µg/ mL) was added as an internal standard. The samples were first agitated at 250 rpm and 80 °C for 10 min and then transferred into a heatex stirrer (set at 1000 rpm and 80 °C) where a solid phase microextraction (SPME) fiber, constituted of Divinylbenzene/ Carbon-Wide Range /Polydimethylsiloxane (DVB/C-WR/PDMS), was introduced into the headspace to adsorb the volatile and semi volatile compounds for 20 min. The SPME fiber was then placed in the gas chromatography’s inlet and allowed to desorb for 1 min.

The gas chromatography mass spectrometer (GC-MS) system used comprised of an AOC6000 autosampler, a 2030 Shimadzu gas chromatograph and a TQ8050NX triple quadrupole mass spectrometer (Shimadzu, Japan). The mass spectrometer was tuned according to the manufacturer’s recommendations using tris-(perfluorobutyl)-amine (CF43). GC-MS was performed on a 30 m GLC Sciences InertCap Pure-WAX column with 0.25 mm internal diameter column and 0.25 μm film thickness. The inlet was set at 250 °C, the mass spectrometer (MS) transfer line at 250 °C and the ion source adjusted to 200 °C. Helium was used as the carrier gas at a flow rate of 1 mL/min. The analysis was performed under the following oven temperature program; 50 °C start temperature, hold for 5 min, followed by a 10 °C/min oven temperature ramp to 250 °C with a following final hold for 10 min. The MS was operated in electron ionisation and MRM (Multiple reaction monitoring) mode. Targeted GC-MS analysis was completed using the Shimadzu Smart Metabolite Database (v1; which covers 496 volatiles, where each target is comprised of a quantifier and qualifier MRM transition. The Resultant data was processed using the Shimadzu LabSolutions Insight software (v4.0), where peak integrations were visually validated and manually corrected where required. 98 annotated metabolites were identified across all groups. All data were analysed in MetaboAnalyst 5.0.

### RNA-sequencing

Total RNA was extracted from crushed fresh frozen lung tissue using a RNeasy Plus kit (Qiagen, Germany) according to manufacturer’s instructions, which was then used for bulk RNA sequencing by the Australian Genome Research Facility (AGRF, Melbourne, Australia). Briefly, the purity and integrity of the RNA was first assessed, followed by library construction with a TruSeq Stranded Total RNA kit (Illumina, San Diego, California, US). Twenty million 150-bp paired end reads were performed on the Illumina NovaSeq 6000 platform, and primary sequence data was then generated with the Illumina DRAGEN BCL Convert 07.021.645.4.0.3 pipeline. The raw sequencing data was trimmed to remove low-quality reads using Trim Galore. The cleaned sequence reads were then aligned against the Mus musculus genome (Build version mm39). The STAR aligner (v2.3.5a) was used to map reads to the genomic sequences to generate the raw gene counts.

### Differentially expressed genes

EdgeR version 3.38.4 was used to identify differentially expressed genes (DEGs) between different groups of comparison. The default trimmed mean of M-values (TMM) normalisation method from EdgeR was used to normalise the counts between samples. A generalised linear model was then used to quantify the differential expression between the groups. DEGs were defined as genes with |logFC| ≥ 1 and false discovery rate (FDR) < 0.05. Visualisation of DEGs on Venn diagram and heatmap was carried out using the R packages ‘ggplot2’, ‘eulerr’ and ‘ComplexHeatmap’. A full list of DEGs is attached in the online file.

### Pathway analysis of DEGs

Gene Ontology (GO), Reactome and Kyoto Encyclopedia of Genes and Genomes (KEGG) pathway analysis of DEGs were conducted using R package ‘clusterProfiler’. Reactome pathways and KEGG pathways with *p*-value < 0.05 and false discovery rate (FDR) < 0.05 were considered significantly enriched. The results were visualized in dot plots using R package ‘ggplot2’.

### Statistical analysis

Statistical analyses were performed with GraphPad Prism 9.0 and graphical data are presented as mean ± SEM. Kolmogorov-Smirnov tests were performed to confirm the normal distribution of the data and parametric tests were subsequently used for all analysis. 2-way ANOVA was performed with Tukey’s or Dunnett’s multiple comparisons post-hoc test where appropriate. Statistical significance is declared where *p* < 0.05 and is indicated with an asterisk (*). (**), (***), (****) are used to indicate p values that are less than 0.01, 0.001, and 0.0001 respectively. All RNAqseq data analyses and visualisation were conducted using R version 4.3.0.

## Results

### CS and HDM co-exposure augmented pulmonary inflammation in C57BL/6 mice

**Fig. 1:**
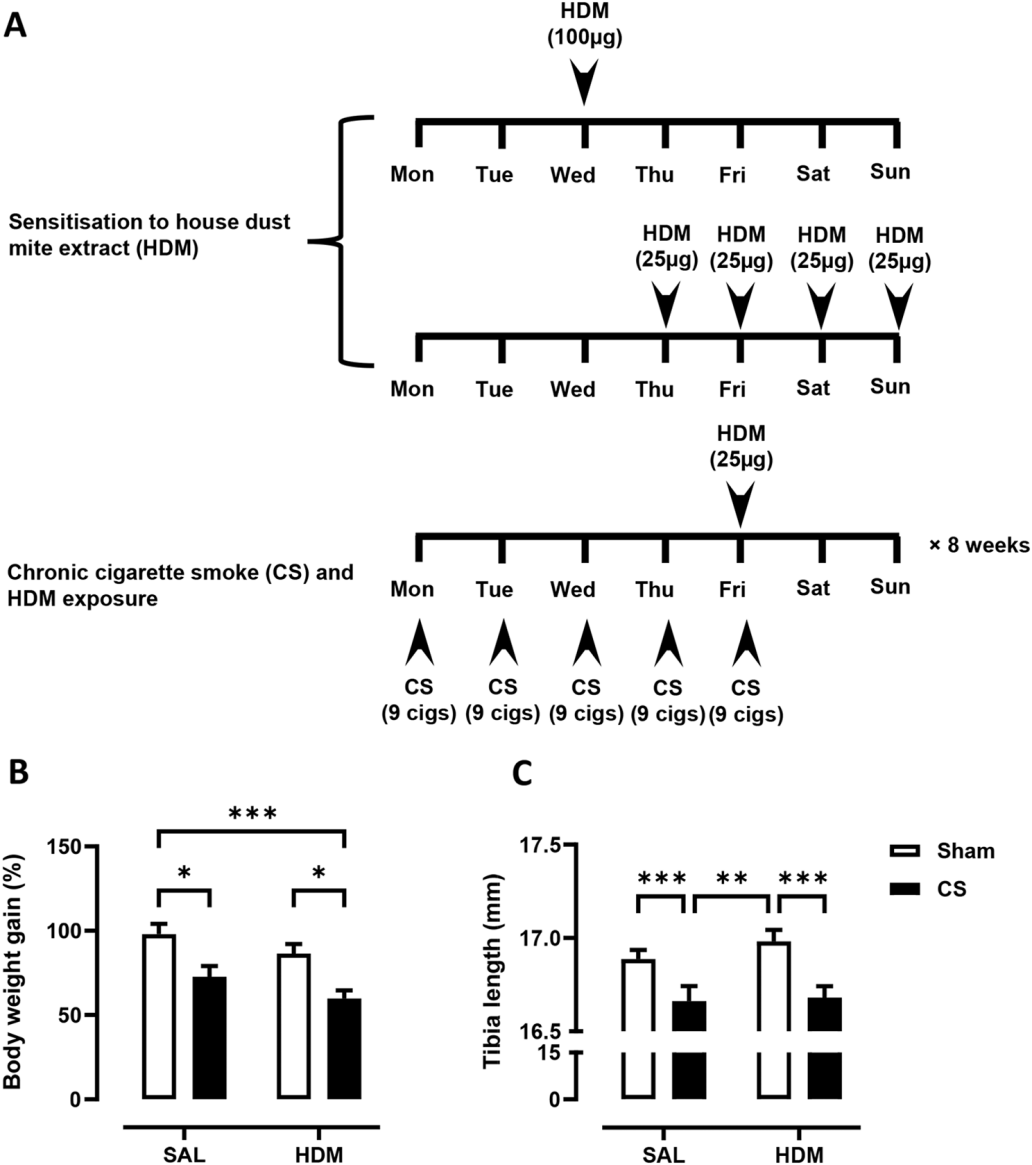
(**A**) 3-week-old C57BL/6 mice were exposed to house dust mite extract (HDM) sensitisation and challenge over 2 weeks, followed by 8-weeks of cigarette smoke (CS) and HDM exposure. Control mice received saline (SAL) and exposed to room air (Sham). The growth of mice, as measured by (**B**) body weight gain and (**C**) tibia length was determined. For each biological group, *n* = 8. *p < 0.05; **p < 0.01; ***p < 0.001, 2-way ANOVA with multiple comparisons across all groups and Tukey’s post-hoc test

**Fig. 2:**
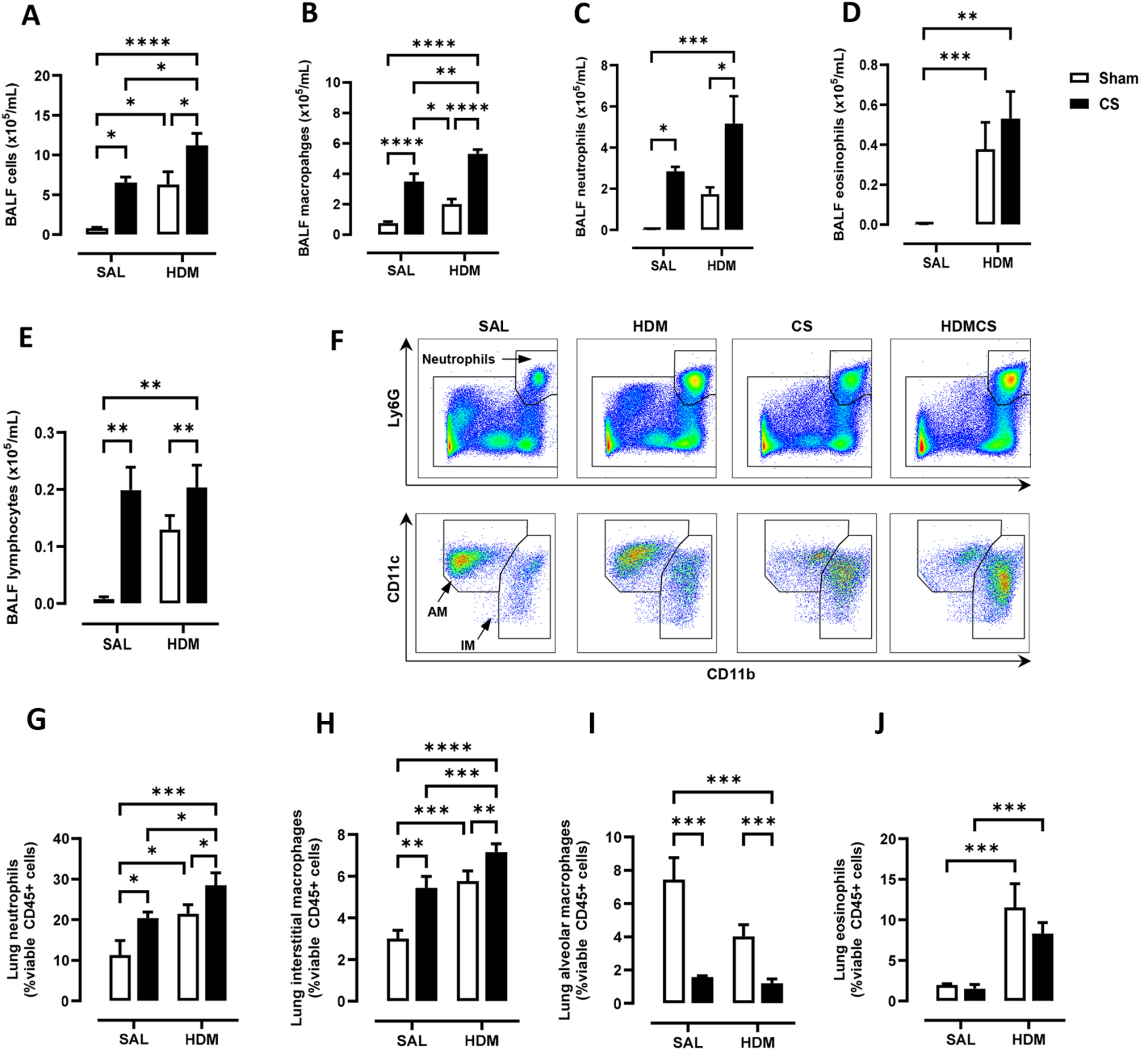
Chronic house dust mite (HDM) and cigarette smoke (CS) exposure induced airway inflammation as determined by (**A**) total bronchoalveolar lavage fluid (BALF) cells. Total (**B**) BAL macrophages, (**C**) neutrophils, (**D**) eosinophils and (**E**) lymphocytes were also increased, as measured by differential counts. Flow cytometry was performed on lung tissue to track myeloid cell populations with representative plots shown in (**F**). Lung neutrophils (**G**), interstitial macrophages (IM) (**H**), alveolar macrophages (AM) (**I**) and eosinophils (**J**) were analysed and presented as percentages over CD45 positive cells. For each biological group, *n* = 6. *p < 0.05; **p < 0.01; ***p < 0.001, ****p < 0.0001 2-way ANOVA with multiple comparisons across all groups and Tukey’s post-hoc test

Young C57BL/6 mice at 3-week of age were first sensitised and challenged with HDM to establish allergic asthma, followed by 8 weeks of exposure to CS and HDM into adulthood, as summarised in Fig. 1A. Over the course of the experiment, mice exposed to CS gained significantly less body weight than control mice with shorter tibia length (Fig. 1B), indicating that mice exposed to CS were physically smaller. These data are consistent with the clinical observation that CS can decrease body mass index and height in males [24]. Next, airway inflammation was measured in the BAL compartment. A 5-fold increase in macrophages, as well as significant infiltration of neutrophils and lymphocytes was observed in CS exposed mice (Fig. 2B, C, E). HDM treatment caused a comparable increase in total BAL cells with evident infiltration of eosinophils (Fig. 2A &D). CS and HDM co-exposure resulted in almost 2-fold increase in total BAL cells compared to CS or HDM alone (Fig. 2A), driven by expansion of multiple leukocytes including macrophages, neutrophils, eosinophils and lymphocytes (Fig. 2B-E). Flow cytometry was then employed to analyse myeloid cell populations in lung tissue using the gating strategy illustrated in Figure S1 as previously described [25]. HDM and CS treatment alone induced approximately a 2-fold increase in neutrophils (Fig. 2F and G) and CD11b^+^ interstitial macrophages (Fig. 2H) in lung tissue, which were further exacerbated in the HDM-CS group. HDM but not CS markedly increased lung eosinophils and this increase was retained in mice with HDM-CS exposure (Fig. 2J). CS but not HDM caused a reduction of alveolar macrophages, which was also observed in mice treated with CS and HDM (Fig. 2I). Flow cytometry was employed to analyse lymphoid cell populations in the local lung tissue and the systemic spleen compartment, and the gating strategy is illustrated in Figure S2A. Lung lymphocytes were enriched for CD4 rather than CD8 T cells in HDM exposed mice, although CD4 T cell enrichment was attenuated with HDM and CS co-exposure (Figure S2B,C). Lung CD4 T cells exhibited a terminally differentiated effector memory (T_EM_) cell phenotype in all treated groups compared to sham, although HDM-exposed mice had the highest levels (Figure S2D). An increase in non-naïve CD4 T cells is consistent with the dominant T helper 2 response seen in response to an allergen that would be expected with HDM treatment. Spleen samples revealed a marked reduction in the number of CD4 and CD8 T cells with CS exposure, regardless of HDM treatment (Figure S2E,F), which indicates that CS exposure may influence lymphopoiesis, apoptosis or trafficking into the spleen.

### CS-induced emphysema was exacerbated in mice exposed to HDM aeroallergen

**Fig. 3:**
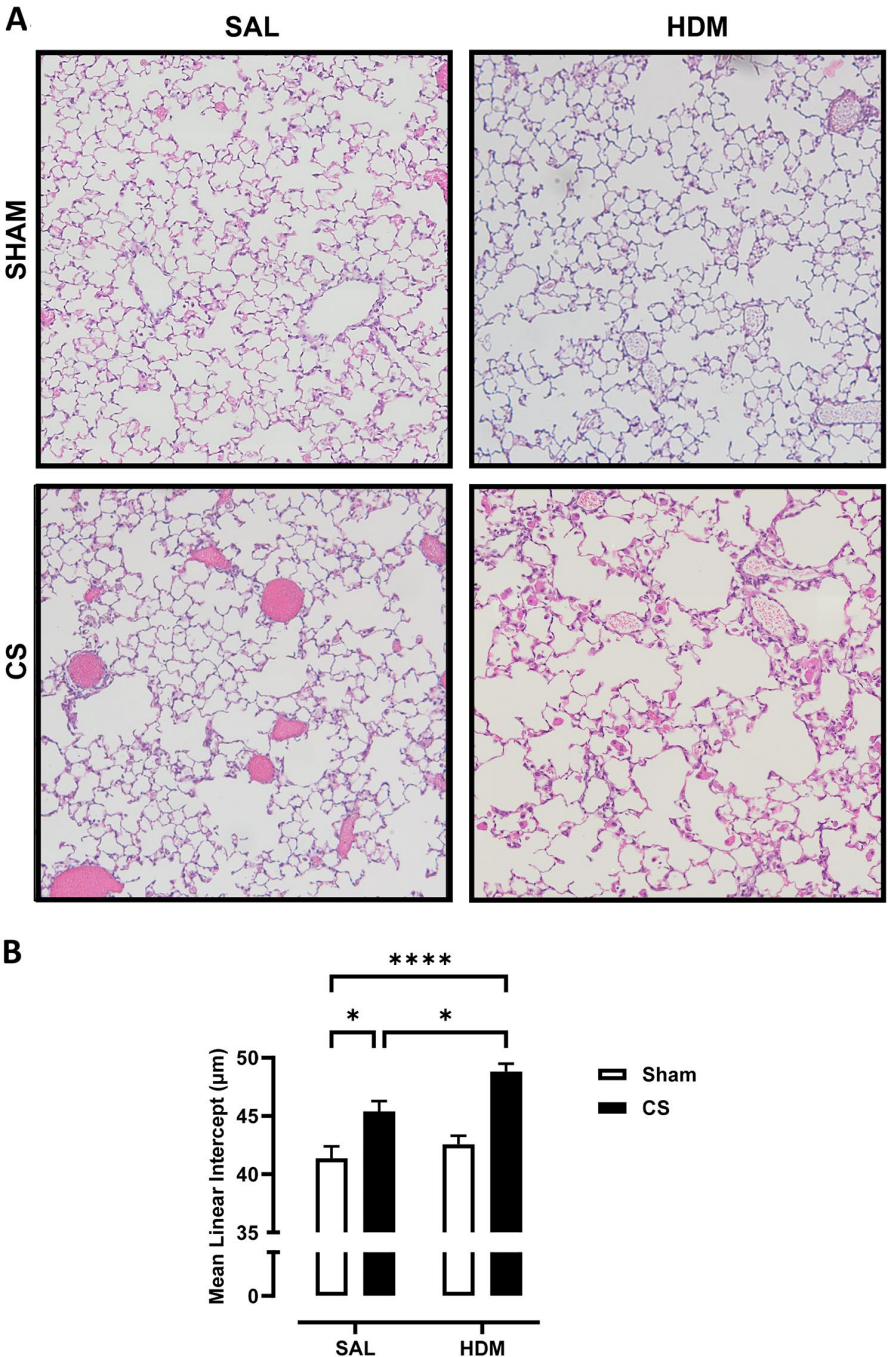
(**A**) Representative images of haematoxylin and eosin (H&E) stained lung sections illustrating emphysematous changes in the lung parenchyma. Chronic CS exposure induced air space enlargement that was enhanced in HDM-CS exposed mice, as measured by (**B**) the mean linear intercept (Lm). For each biological group, *n* = 8. *p < 0.05; ****p < 0.0001 2-way ANOVA with multiple comparisons across all groups and Tukey’s post-hoc test

To assess whether 2-month CS exposure induced emphysema and whether this was altered by early life exposure to HDM allergen, mean linear intercept (L_m_) was performed on H&E-stained whole lung sections to quantify the airspace enlargement or emphysema. Representative images of lung parenchyma are shown in Fig. 3A. Lung sections of mice not exposed to CS (Sham-SAL and Sham-HDM) showed well-structured and intact alveoli, while CS exposure resulted in moderate disruption of alveolar septum. This observation was more evident in HDM and CS exposed mice with extensive loss of alveolar structures. As shown in Fig. 3B, the baseline L_m_ was measured to be 41.4 μm. CS significantly increased L_m_ to 45.4 μm, while HDM treatment did not result in any changes to L_m_ (42.6 μm). Importantly, CS and HDM co-exposure resulted in a marked increase in L_m_ (48.8 μm) which was significantly higher than the control, CS-only, and HDM-only groups. This demonstrates that chronic CS resulted in airspace enlargement, a defining feature of emphysema, and the extent of emphysema was further increased in mice exposed to allergen and CS.

### Transcriptomic analysis revealed common and distinct gene clusters in mice exposed to HDM and CS

**Fig. 4:**
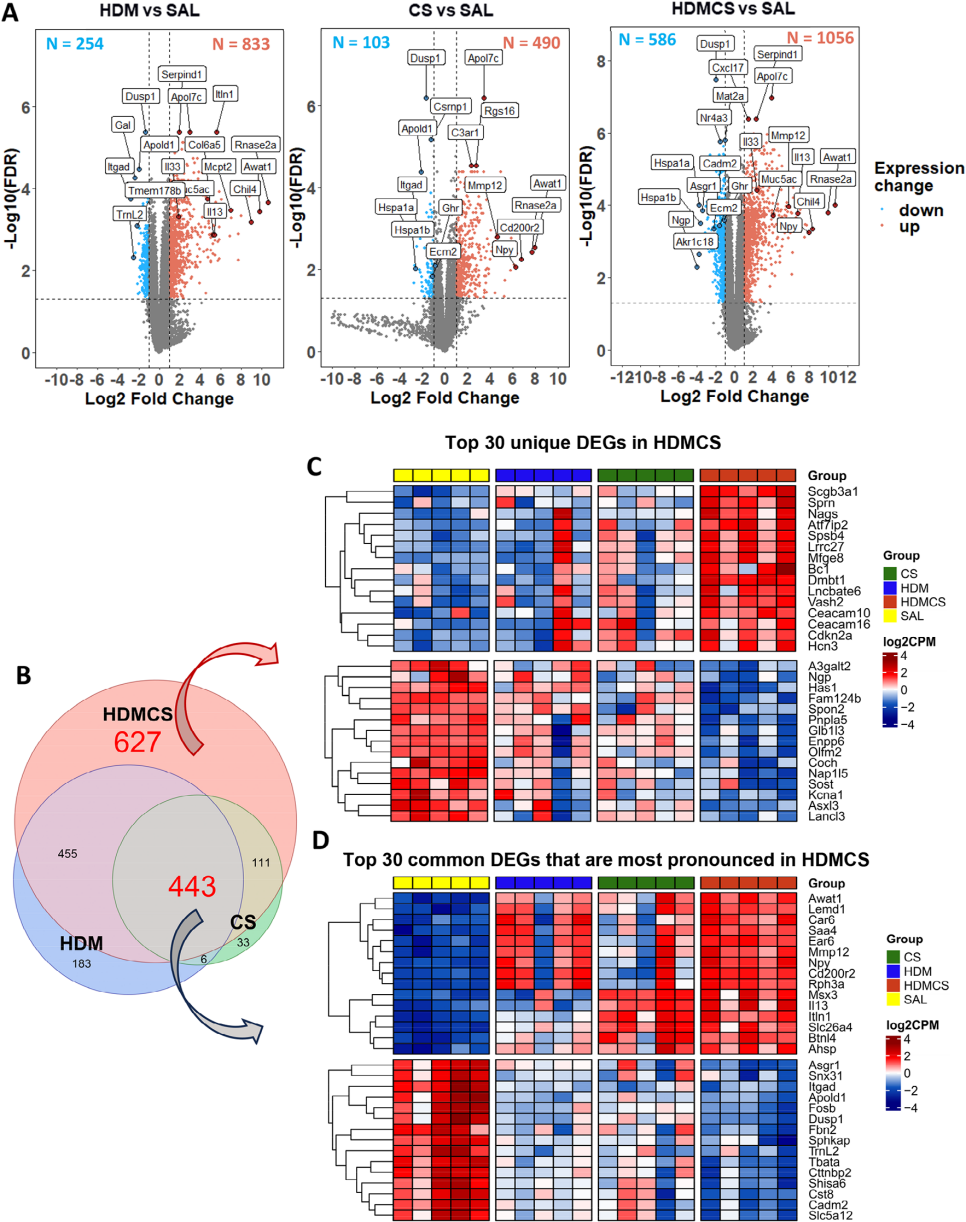
RNAseq was performed on lung tissue and differentially expressed genes (DEGs) are visualized in volcano plots (**A**), which identify upregulated DEGs (in red dots) and downregulated DEGS (in blue dots) compared to SAL group. Immunoglobulin gene segments, as well as predicted and pseudo genes are not included. Top genes ranked by fold change or FDR are highlighted in each plot. In addition, gene markers associated with type 2 inflammation (*Mcpt2*, *Il13*, *Il33*) and lung remodelling (*Col6a5*, *Muc5ac*) are highlighted in the HDM vs. SAL plot; gene markers associated with emphysema development (*Mmp12*) and lung remodelling (*Ecm2*) are highlighted in the CS vs. SAL plot. These featured genes are also highlighted in the HDM-CS vs. SAL plot. Venn diagram (**B**) further demonstrates among all the DEGs, 627 DEGs are unique to HDM-CS group and 443 DEGs are common to all three experimental groups. (**C**) Within the 627 DEGs unique to HDMCS group, heatmap shows the expression level of the top 15 upregulated DEGs (upper panel) and top 15 downregulated DEGs (lower panel) ranked by fold change. (**D**) Among the 443 common DEGs, the heatmap further reveals the expression levels of the top 15 upregulated (upper panel) and downregulated (lower panel) DEGs that are most pronounced in HDM-CS treated mice (higher than HDM or CS. For each biological group, *n* = 5

To investigate the molecular mechanisms underpinning the inflammatory response and lung remodelling, bulk RNAseq analysis was performed on mouse lungs. As demonstrated in the volcano plots in Fig. 4A, relative to the control group, 1087 genes were differentially expressed (DEGs) in HDM treated mice, with 833 upregulated and 254 downregulated. The most upregulated genes include eosinophil marker *Rnase2a*, lipid metabolism enzymes *Awat1* and *Apoldl*, type 2 inflammation and macrophage alternative activation (M2) associated gene *Chil4 (Ym2)*, endogenous thrombin inhibitor *Serpind1*, and immune cell receptor *Itln1.* In addition, classic type 2 inflammatory markers *Il33* and *Il13*, mucin gene *Muc5ac*, fibrosis-related gene *Col6a5*, and mast cell marker *Mcpt2* were upregulated. Top downregulated genes include vascular gene *Apold1*, neurotransmitter *GAL*, and integerin gene *Itgad*. In CS-exposed mice, 490 DEGs were upregulated, including COPD susceptible gene *Mmp12*, smoking related gene *Npy*, apolipoprotein *Apol7c*, and complement component C3a *C3ar1*. Extracellular matrix gene *Ecm2*, and stress related heat shock proteins genes (*Hspa1a*, *Hspa1b*), and growth hormone receptor *Ghr* were amongst the top down-regulated genes. The HDM-CS group exhibited a significant increase in the number of DEGs (1642 genes), indicating that more substantial biological changes occur under combined exposure. The DEGs from the HDM and CS individual groups were also detected in the HDM-CS group, suggesting conservation of these transcriptomic responses. Of interest, *Cxcl17*, a monocyte/macrophage chemoattractant was highly upregulated in HDM-CS treated mice.

**Fig. 5:**
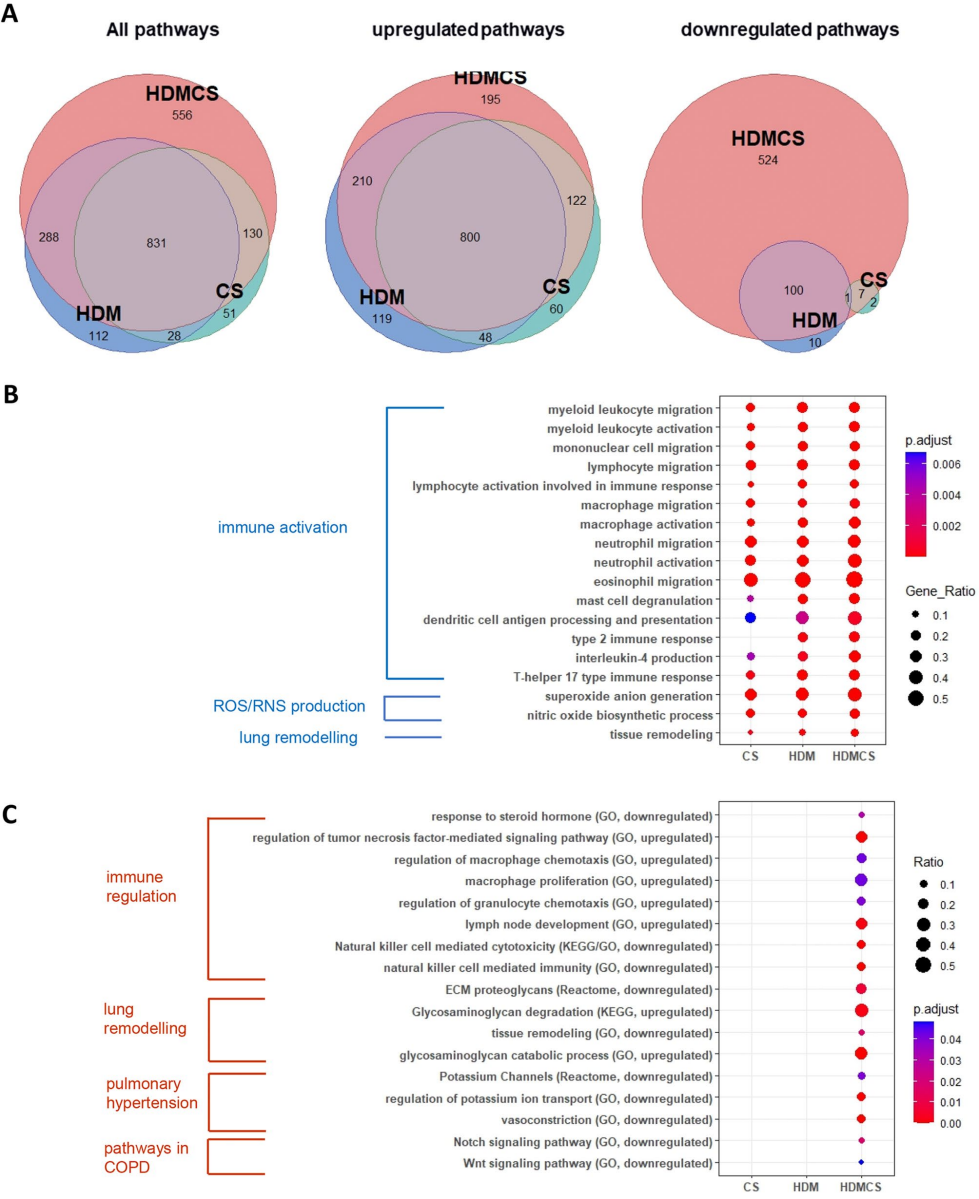
Gene set enrichment analysis (GSEA) of KEGG, Reactome and GO database was performed on the DEGs. Pathway overlapping was visualized in Venn diagrams (**A**) where pathways that were common to all three disease models and pathways unique to HDM-CS model were denoted. Dot plots showed the top GO terms that were common to all three disease models (**B**), as well as GO, KEGG and Reactome terms derived from the DEGs exclusively found in HDM-CS treated mice (**C**). For each biological group, *n* = 5

The Venn diagram further illustrates DEGs unique to the HDM-CS group (627 DEGs) and DEGs common to all three experimental groups (443 DEGs) (Fig. 4B). Heatmap in Fig. 4C shows the group differences for the top 30 unique DEGs unique to HDM-CS, with upregulated genes indicating involvement in angiogenesis (*Vash2*), cell adhesion (*Ceacam10*, *Ceacam16*), and cell cycle regulation (*Cdkn2a*). Downregulated genes suggest roles in bone development (*Sost*), lipid metabolism (*Pnpla5*), and ion channel activity (*Kcna1*). Heatmap in Fig. 4D details the top 30 most pronounced DEGs in HDM-CS, with upregulated genes including COPD marker *Mmp12*, type 2 marker *Il13*, eosinophil gene *Car6*, acute reactant *Saa4*, and *Npy*. Downregulated genes include those in ECM formation (*Fbn2*), protease inhibition (*Cst8*), and vascular regulation (*Apold1*). RTqPCR analysis confirmed the gene expression of M2 macrophage markers and Th2/Th17 cytokines, revealing the highest expression levels in the HDM-CS group (Figure S3). Next, GO, Reactome and KEGG database enrichment analysis and functional annotation were performed on the DEGs. As seen in the Venn diagrams in Fig. 5A, both overlapping pathways and distinct pathways were identified in the HDM-CS group. Common pathways driven by CS or HDM alone are conserved in the HDM-CS group, which include immune activation, reactive oxygen/nitrogen specifies (ROS/RNS) production and lung remodelling (Fig. 5B). Distinct pathways that were only detected in HDM-CS treated mice were identified to be involved in immune regulation, lung remodelling, pulmonary hypertension and Notch/Wnt signaling that has been implicated in COPD and other respiratory diseases (Fig. 5C).

### CS and HDM exposure altered the volatile organic compound composition in BALF

**Fig. 6:**
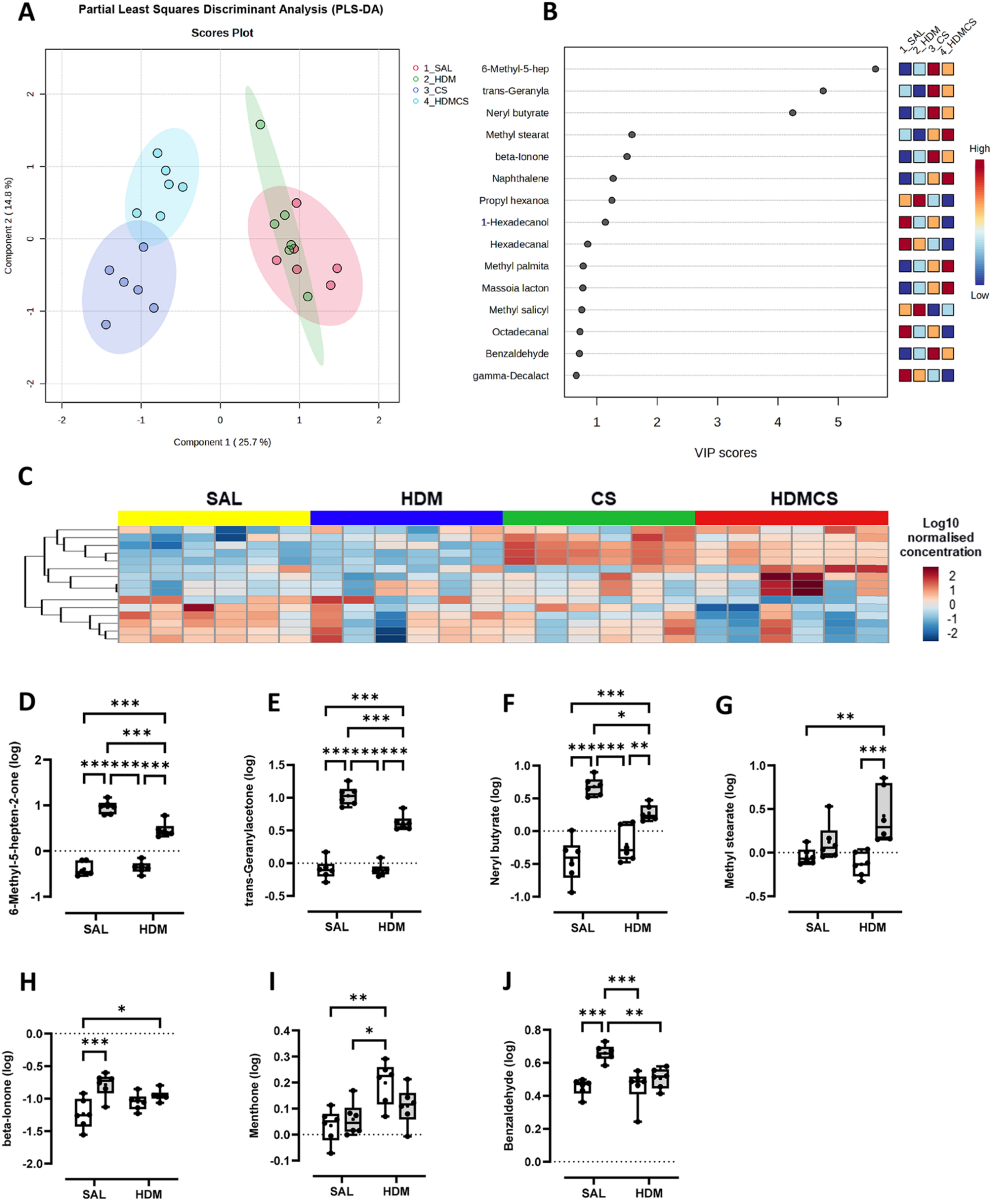
Volatile metabolites were measured in the bronchoalveolar lavage fluid (BALF) using gas chromatography-mass spectrometry, identifying 92 annotated metabolites. (**A**) Partial Least Squares Discriminant Analysis (PLS-DA) plot demonstrating the different clustering across treatment groups. (**B**) Top 15 features from the PLS-DA analysis were presented with variable importance in projection (VIP) scores and also visualised in (**C**) heat map using median normalized, log transformed values. (**D** − **J**) All 7 VOCs that were detected with statistical difference in multivariant analysis were presented. *p < 0.05; **p < 0.01; ***p < 0.001, ****p < 0.0001 2-way ANOVA with multiple comparisons across all groups and Tukey’s post-hoc test. For each biological group, *n* = 6

Trace volatile organic compounds (VOCs) in EBC and BAL fluid can be altered in respiratory diseases, which hold promise for disease phenotype and biomarker identification. To determine whether the composition of VOCs was altered in our disease models, BAL fluid was analysed by gas chromatography and mass spectrometry (GC-MS), which detected a total of 92 metabolites from 500 compounds screened. As demonstrated in the Partial Least Squares Discriminant Analysis (PLS-DA) plot (Fig. 5A), whilst HDM group largely overlapped with SAL group, CS and HDM-CS groups clustered away from the SAL and HDM groups, suggesting CS had a profound effect on volatile metabolite composition. HDM-CS was also distinct from the CS group, suggesting an interaction between CS and HDM. The variable importance in projection (VIP) was used as a determinant measure in the PLS-DA, and the VIP scores of the top 15 VOCs were presented in Fig. 6B and also visualised in the heatmap in Fig. 6C. In all 92 VOCs annotated, 7 VOCs were further identified to be altered with statistical difference across the experimental groups (Fig. 6D-J). 5 out the 7 VOCs were increased by CS and 4 remained elevated in HDM-CS group, namely 6-Methyl-5-hepten-2-one, trans-Geranylacetone, Neryl butyrate, and beta-lonone, all of which were previously detected in human bodily fluids [26]. Interestingly, the levels 4 CS-induced VOCs were significantly lower in the HDM-CS group in comparison to CS alone.

## Discussion

Epidemiological studies indicate that childhood asthma increases the risk of COPD later in life, especially in smokers [8, 27]. Our current study investigated this relationship in an experimental setting by interrogating whether early life allergic airway disease triggered by HDM exposure accelerates emphysema induced by CS in mice. This study shows that mice with a pre-existing mild asthma phenotype, developed worse emphysema and lung remodelling after chronic and concurrent exposure to CS and HDM compared to mice that were only exposed to CS. This notion was supported by the increase in Lm values, downregulation of ECM gene *Ecm2* and cell adhesion molecule 2 (*Cadm2*), and upregulation of mucin gene *Muc5ac*. Our findings imply that children with persistent asthma who start smoking at a young age are at an increased risk of developing fixed airflow obstruction and COPD in adulthood, consistent with clinical observations [9].

Mechanistically, our RNAseq and qPCR analysis revealed emphysema in CS exposed mice was accompanied with an upregulation of COPD susceptible gene *Mmp-12*, a well-recognised genetic factor for COPD both in humans and in pre-clinical models [28]. Of greater interest, dual exposure to CS and HDM resulted in more extensive airspace enlargement and lung remodelling, where *Mmp12* expression was further increased, identifying MMP-12 as a candidate mediator responsible for the aggravated lung pathology. In line with this finding, *Mmp12* single-nucleotide polymorphisms (SNPs) correlation analysis conducted on the CAMP clinical cohort and other COPD cohorts also identified that a minor SNP allele in *Mmp12* was associated with lung function in children with asthma and in adults who smoke [28]. In our study, the heightened *Mmp12* expression in HDM-CS mice was detected alongside the activation of canonical type 2 immune response consisting of elevated transcript levels of *Il-13, Il-33* and *Il-4*, as well as a gene signature suggestive of M2/alternatively activated macrophages. IL-13 is capable of promoting M2 polarisation and MMP-12 production in macrophages and in mice, where over-expression of IL-13 was found to induce emphysema that was dependent on MMPs, with eosinophils identified to be a rich source of IL-13 [29, 30]. Likewise, IL-33 and IL-4 can also facilitate the emergence of M2 macrophages and MMP-12 induction [31, 32]. Furthermore, IL-33 levels were reported to be elevated in ACO patients, and intratracheal treatment with recombinant IL-33 resulted in expansion of interstitial macrophages in the lungs of mice [33]. It should be noted that while type 2 inflammation may be important in accelerating expansion of pathogenic macrophages, it alone is insufficient to cause emphysema in our model and this process is still dependent on chronic exposure to CS. Increased expression of MMP-12 is known to degrade elastin fibres in the lungs, producing chemotactic elastin fragments that create a positive feedback loop, further recruiting macrophages that are derived from the peripheral blood monocytes. Consistent with expansion of interstitial lung macrophages and MMP-12 expression, was the hyper-activation of the ‘mononuclear cell migration’ pathway in HDM-CS mice as determined in our RNAseq analysis.

The analysis of volatiles in the BAL fluid shows that CS drove a significant shift in the composition of VOCs, while HDM alone had limited effect. Interestingly, when CS was combined with HDM, the VOC profile became distinct from CS. In particular, a group of volatiles, including 6-Methyl-5-hepten-2-one, trans-Geranylacetone, Neryl butyrate, Methyl stearate, beta-lonone and Benzaldehyde were found to be significantly increased with CS, among which Benzaldehyde in exhaled breath condensate (EBC) has already been proposed as biomarker of COPD [34]. Four of the VOCs were markedly reduced with HDM co-treatment relative to CS alone and are known to be additive/flavouring constituents within cigarette-related products [35, 36]. The reason for reduced levels is unknown but may indicate that mice exposed to both CS and HDM acutely inhale less CS than mice exposed to CS alone. Indeed, it has been found that acute bronchoconstriction occurs during cigarette smoking in asthmatics [37], which may limit the amount of smoke entering the distal airways and alveoli.

In summary, our study identified early-life exposure to aeroallergen accelerated the development of CS-induced emphysema later in life. It was further shown that the pre-existing type 2 immune signature upon the commencement of CS exposure likely contributed to the onset of emphysema with increased production of MMP-12 and recruited pathogenic lung macrophage populations such as interstitial macrophages. This novel model of can be used to further investigate the pathophysiology of COPD to develop endotype-specific treatments.

### Electronic supplementary material

Below is the link to the electronic supplementary material.

Supplementary Material 1

Supplementary Material 2

Supplementary Material 3

## Data Availability

A full list of DEGs generated from the RNAseq analysis is attached in the online file.

## Abbreviations

AAM: Alternatively activated macrophages
ACK: Ammonium-chloride-potassium
ACO: Asthma-COPD overlap
ANOVA: Analysis of Variance
BAL: Bronchoalveolar lavage
BALF: Bronchoalveolar lavage fluid
CAMP: Childhood Asthma Management Program
CD: Cluter of Differentiation
cDNA: Complementary deoxyribose nucleic acid
COPD: Chronic obstructive pulmonary disease
CS: Cigarette smoke
DEGs: Differentially expressed genes
EBC: Exhaled breath condensate
FDR: False discovery rate
FEV1: Forced expiratory volume (in 1 s)
Fizz1: Resistin-like alpha
FVC: Forced vital capacity
GAPDH: Glyceraldehyde-3-phosphate dehydrogenase
GC-MS: Gas chromatography and mass spectrometry
GO: Gene Ontology
H&E: Haematoxylin and eosin
HBSS: Hank’s buffered salt solution
HDM: House dust mite extract
HRQoL: Health-related quality of life
IL-: Interleukin-
KEGG: Kyoto Encyclopedia of Genes and Genomes
Lm: Mean linear intercept
MMP: Matrix metalloproteinase
NBF: Neutral buffered formalin
PCR: Polymerase chain reaction
PLS-DA: Partial Least Squares Determinant Analysis
RNA: Ribonucleic acid
RNAseq: RNA sequencing
RNS: Reactive nitrogen species
ROS: Reactive oxygen species
RTqPCR: Reverse transcription qualitative real-time PCR
SAL: Saline
T_EM_: Effector memory T cell
Th2: T helper 2
TMM: Trimmed mean of M-values
VIP: Variable importance in projection
VOC: Volatile organic compound
