# Supplementary material for: Early-life house dust mite aeroallergen exposure augments cigarette smoke-induced myeloid inflammation and emphysema in mice
Source: Respir Res. 2024 Apr 13;25:161. doi: 10.1186/s12931-024-02774-6 (PMC11016214; doi:10.1186/s12931-024-02774-6)
Supplement: Supplementary file 1 — Supplementary Material 1 [file 12931_2024_2774_MOESM1_ESM.docx]

**Supplement Figure legends.**

**Figure S1.** Gating strategy for analysing lung myeloid cells by flow cytometry. Viable cells are first gated using Fixable Live/Dead dye (Cy7) versus autofluorescence. Single cells are then gated, followed by gating Ly6G+ neutrophils within the CD45+ population. CD49b+ NK cells are gated among the non-neutrophil cells. Non-myeloid cells, which are double negative for CD11b and CD11c, are excluded. Eosinophils are identified based on the absence of CD11c and high expression of SiglecF. Macrophages are selected based on their expression of MerTK and CD64 and are further subdivided into tissue-resident alveolar macrophages (AMs) with high CD11c expression and negative CD11b expression, as well as interstitial macrophages (IMs) with high CD11b expression. Ly6C^high^ monocytes are determined within the non-macrophage population based on their high Ly6C expression.

**Figure S2.** Impact of chronic HDM and CS exposure on lymphoid cells. (A) Gating strategy for defining T cell subsets. The frequency of (B) CD4 and (C) CD8 T cells in lung leukocytes, and (D) the frequency of CD4 T cells that are T_EM_ cells. The number of (E) CD4 and (F) CD8 T cells in the spleen. For each biological group, n = 8. * p < 0.05; ** p < 0.01; *** p < 0.001, **** p < 0.0001. 2-way ANOVA with Tukey’s multiple comparisons.

**Figure S3.** RT-qPCR analysis on lung expression of (A) *Mmp-12*, (B) *Retnla*, (C) *Arg1*, (D) *Chil3*, (E) *Il-33*, (F) *Il-13*, (G) *Il-5* and (H) *Il-17* gene. For each biological group, n = 8. * p < 0.05; ** p < 0.01; *** p < 0.001, **** p < 0.0001. 2-way ANOVA with Tukey’s multiple comparisons.
