# Supplementary material for: Early-life house dust mite aeroallergen exposure augments cigarette smoke-induced myeloid inflammation and emphysema in mice
Source: Respir Res. 2024 Apr 13;25:161. doi: 10.1186/s12931-024-02774-6 (PMC11016214; doi:10.1186/s12931-024-02774-6)
Supplement: Supplementary file 2 — Supplementary Material 2 [file 12931_2024_2774_MOESM2_ESM.pptx]

## Slide 1
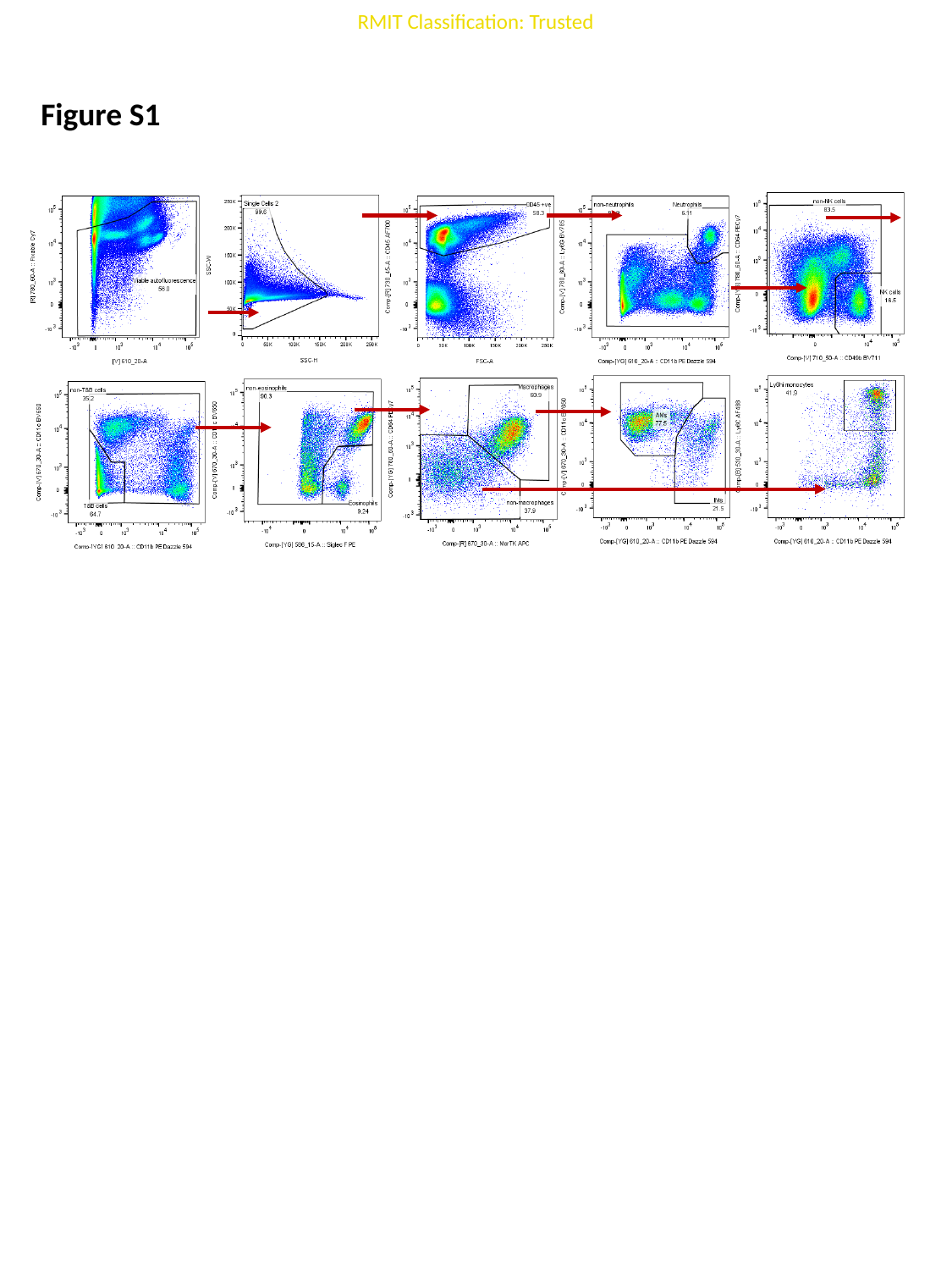

Figure S1

## Slide 2
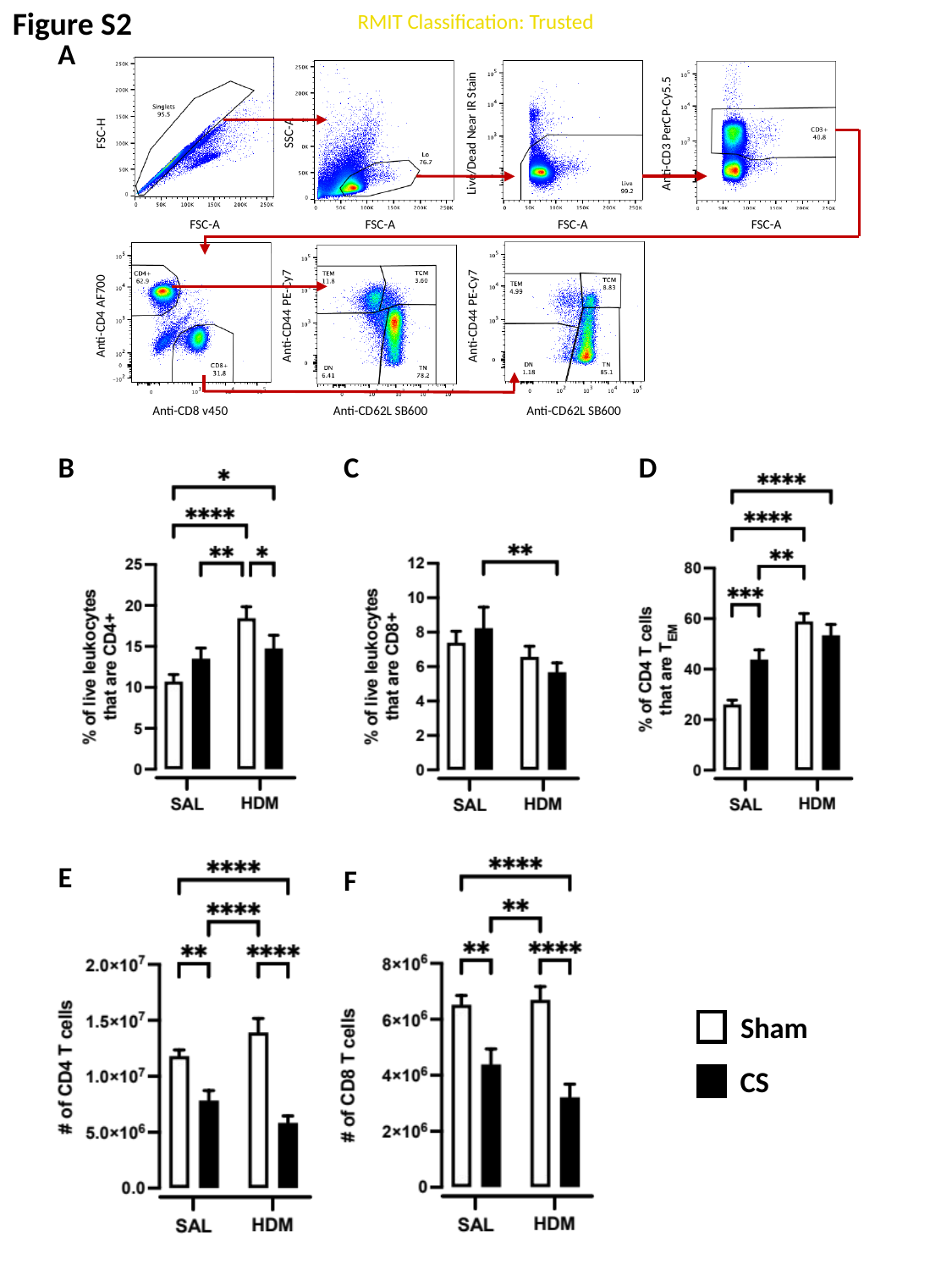

Figure S2
A
FSC-H
Live/Dead Near IR Stain
Anti-CD3 PerCP-Cy5.5
SSC-A
FSC-A
FSC-A
FSC-A
FSC-A
Anti-CD4 AF700
Anti-CD44 PE-Cy7
Anti-CD44 PE-Cy7
Anti-CD8 v450
Anti-CD62L SB600
Anti-CD62L SB600
B
C
D
E
F
Sham
CS

## Slide 3
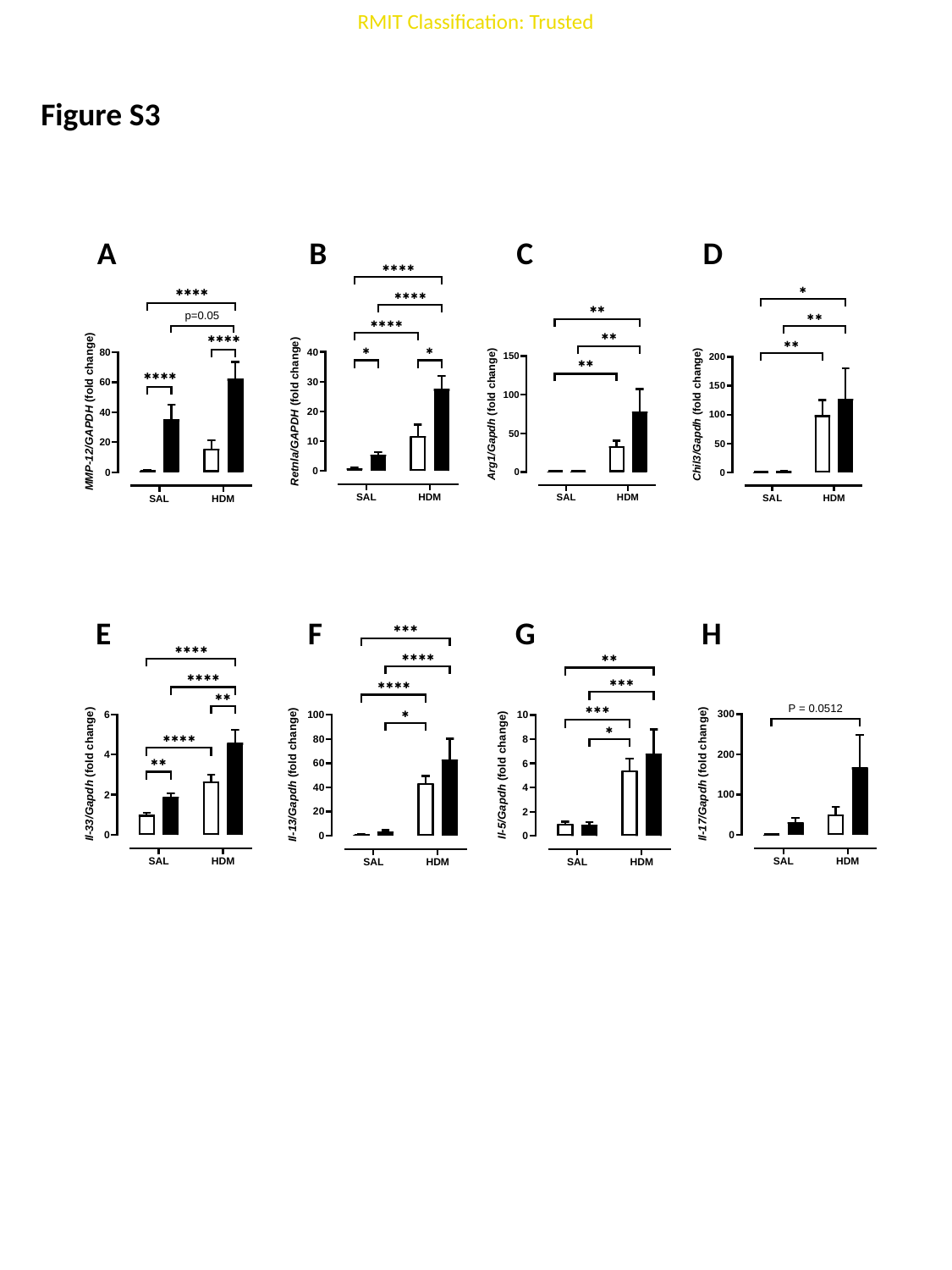

Figure S3
A
B
C
D
E
F
G
H
